# Bioinformatics Analysis of the Glutamate-Gated Chloride Channel Family in *Bursaphelenchus xylophilus*

**DOI:** 10.3390/ijms26083477

**Published:** 2025-04-08

**Authors:** Haixiang Li, Rui Wang, Jialiang Pan, Jie Chen, Xin Hao

**Affiliations:** 1 Key Laboratory of National Forestry and Grassland Administration on Prevention and Control Technology of Pine Wilt Disease, Anhui Academy of Forestry, Hefei 230088, China; 18098051238@163.com (H.L.); panjialiang1987@126.com (J.P.); 2 Laboratory of Forest Disaster Warning and Control in Yunnan Province, Southwest Forestry University, Kunming 650224, China; 15721824391@163.com

**Keywords:** *Bursaphelenchus xylophilus*, glutamate-gated chloride channels family, gene identification, gene expression analysis

## Abstract

Glutamate-gated chloride channels (GluCls), a class of ion channels found in the nerve and muscle cells of invertebrates, are involved in vital life processes. *Bursaphelenchus xylophilus*, the pathogen of pine wilt disease, has induced major economic and ecological losses in invaded areas of Asia and Europe. We identified 33 GluCls family members by sequence alignment analysis. A subsequent bioinformatic analysis revealed the physicochemical properties, protein structure, and gene expression patterns in different developmental stages. The results showed that *GluCls* genes are distributed across all six chromosomes of *B. xylophilus*. These proteins indicated a relatively conserved structure by NCBI-conserved domains and InterPro analysis. A gene structure analysis revealed that *GluCls* genes consist of 5 to 14 exons. Expression pattern analysis revealed *BxGluCls* were extensively involved in the development of second instar larvae of *B. xylophilus*. Furthermore, *BxGluCls*15, *BxGluCls*25, and *BxGluCls*28 were mainly associated with the development of eggs of *B. xylophilus*. *BxGluCls*12, *BxGluCls*18, and *BxGluCls*32 were predominantly linked to nematode resistance and adaptation. Investigation the structure and expression patterns of BxGluCls is crucial to understand the developmental trends of *B. xylophilus*. It also helps identify molecular targets for the development of biopesticides or drugs designed to control this nematode.

## 1. Introduction

*Bursaphelenchus xylophilus*, the causal agent of pine wilt disease (PWD), is widely recognized as a critical quarantine pest globally [1]. This pathogen induces rapid wilting and mortality in infected pine, typically resulting in tree death within 2 to 3 months. The swift dissemination of the disease significantly complicates its management and control [2]. By 2024, PWD had spread to 620 counties in 18 provinces of China, posing a severe threat to forest ecosystems and biodiversity [3]. Effective control strategies remain a priority due to the pathogen’s ecological and economic impacts.

Glutamic acid functions as a critical neurotransmitter in the central nervous system of both vertebrates and invertebrates [4]. It mediates nerve signal transmission by interacting with specific glutamate receptors located on the cell membrane. In vertebrates, ligand-gated glutamate receptors facilitate excitatory signaling through cation channel activation. By contrast, in invertebrates, glutamate exhibits dual functionality, serving either as an excitatory or inhibitory neurotransmitter, depending on the receptor subtype and physiological context [5]. A notable feature of invertebrate systems is the presence of glutamate-gated chloride channels (GluCls), which represent a unique subclass of ionotropic receptors within the cysteine-loop superfamily, also referred to as Cys-loop ligand-gated ion channels (Cys-loop LGICs). These channels play a pivotal role in mediating inhibitory neurotransmission [6]. When glutamate binds to ionotropic inhibitory glutamate receptors (iGluRs) in invertebrates, it triggers the opening of GluCls, resulting in chloride ion influx and postsynaptic inhibition. This mechanism highlights the versatility of glutamate as a neurotransmitter and underscores the evolutionary divergence of it signaling pathways across different phyla. The structural and functional diversity of glutamate receptors reflects their crucial roles in synaptic communication. In vertebrates, the primary role of glutamate is excitatory, mediated predominantly through ionotropic receptors such as AMPA, NMDA, and kainate receptors. Conversely, in invertebrates, glutamate’s inhibitory actions via GluCls underscore the adaptation of this amino acid to diverse neuronal functions [7]. The precise regulation of these channels is essential for maintaining synaptic homeostasis and preventing pathological conditions associated with excessive excitation or inhibition.

GluCls have not been detected in vertebrates; rather, they are exclusively present in the nerve and muscle cells of invertebrates, making these ion channels an ideal target for insecticides [8]. In nematodes, including *Caenorhabditis elegans* and *Haemonchus contortus*, GluCls are widely distributed throughout the nervous system [9,10]. These channels play critical roles not only in mediating rapid inhibitory effects within neural cells but also in regulating essential life activities such as movement and swallowing. For insects, GluCls are vital for various physiological functions, including sensation, locomotion, and feeding [11]. In nematodes, GluCls are extensively expressed in both neuronal and muscular tissues. To date, a total of eight distinct GluCls have been identified, each consisting of six *α* subunits, one *β* subunit, and one γ subunit. Interestingly, in insects, only a single GluCl subunit has been characterized thus far [12]. The first insect GluCls were cloned from *Drosophila melanogaster* and exhibited significant homology to both the *α* and *β* subunits of nematode GluCls, specifically those from *C. elegans* [13,14]. This conservation highlights the functional importance of GluCls across diverse invertebrate species, despite their structural variability.

GluCls were reported to be targets of macrolides, indole diterpenoids, and phenyl pyrazoles [15]. The direct action of ivermectin, an antihelminthic drug, on GluCls in locust myofibrils was first demonstrated by Janssen and co-workers [16]. The full-length sequence of the GluCl*α* subunit from *Plutella xylostella* was cloned, followed by a sequence alignment analysis. This analysis demonstrated a 73% sequence similarity between the *P*. *xylostella* GluCl*α* subunit and its orthologs in *Tribolium castaneum* (red flour beetle) and *D*. *melanogaster* (fruit fly). This finding underlines the conservation of GluCls across various insects and nematodes, which is a molecular foundation for developing selective nematicide against *B. xylophilus* [17]. To sum up, due to their exclusive presence in invertebrates and their potential as highly selective targets, GluCls play a crucial role in the quest for neuroactive insecticides with inherent selectivity.

To elucidate the role of GluCls in the development of *B*. *xylophilus*, this study utilized genomic data to identify and bioinformatically analyze members of BxGluCls gene family. Given the critical role of GluCls in nematode physiology and their potential as targets for nematicides, we also explored the possibility that these channels might contribute to drug resistance in *B. xylophilus*. A functional analysis of *BxGluCls* informed the development of innovative biopesticides, which were designed to work in tandem with existing potent drugs. The study commenced with the screening of BxGluCls family members from genomic data, proceeding to analyze their gene structure, physicochemical properties, conserved motifs, and protein structures. Functional predictions were made using molecular docking techniques. Specifically, the main goal of our study was to perform screening of BxGluCls family members from genomic data, analyzing the structure of their encoding genes, and assessing the physicochemical properties, conserved motifs, and protein structures of BxGluCls. Furthermore, we attempted to predict potential drug binding sites on GluCls through molecular docking.

## 2. Results

### 2.1. Identification of BxGluCls

After conducting a BLASTX search in the Wormbase database and validating the conserved domains using Conserved Domains and InterPro, 33 different GluCls were identified in *B. xylophilus*, which were named BxGluCls1 to BxGluCls33, based on their genomic numbering order (Table 1). Then, we compared the protein sequences of GluCls discovered in BxGluCls1 to BxGluCls33 with the sequences of the relevant proteins from other nematodes in order to assess the extent of similarity between these. The comparison revealed that while GluCls in *B. xylophilus* share significant sequence similarity with those from other nematodes, there are notable differences that may reflect species-specific adaptations or functional diversification. These findings highlight the importance of understanding the evolutionary and functional context of GluCls in different nematode species, which could have implications for targeted interventions or therapeutic strategies.

The calculation formula for the E-value is:

E = K × m × n × e^−λS^ (1)

m is the length of the query sequence, n is the total length of all sequences in the database, K and λ are parameters related to the scoring system and the composition of the database, S is the alignment score.

### 2.2. Physicochemical Analysis of BxGluCls

In order to assess the extent of similarity among the different GluCls, we determined their physichochemical properties, including the number of amino acids, molecular weight, isoelectric point (PI), aliphatic index, hydropathicity and instability index. This was accomplished by means of Expasy 3.0 software.

The physicochemical property assay of BxGluCls family members revealed that the average number of amino acids in BxGluCls proteins was 535.33. The average molecular mass of the BxGluCls protein family was 61.63 kDa, with the smallest being BxGluCls8 at 27.29 kDa and the largest being BxGluCls17 at 105.70 kDa. Additionally, the theoretical isoelectric point distribution of the BxGluCls protein family ranged from 4.79 to 9.76. The proteins BxGluCls5, BxGluCls8, BxGluCls22, BxGluCls23, BxGluCls24, and BxGluCls31 are hydrophobic, while all other proteins are hydrophilic. BxGluCls3, BxGluCls5, BxGluCls17, BxGluCls22, and BxGluCls26 were found to be stable, while the remaining proteins were unstable (Table 2).

### 2.3. Phylogenetic and Conserved Motif Analysis of BxGluCls

In order to assess the extent of phylogenetic similarity among the 33 protein sequences, we conducted phylogenetic and conserved motif analyses of BxGluCls. We performed multiple alignments of 33 BxGluCls sequences. The optimal model was chosen according to the maximum likelihood tree. The maximum likelihood evolutionary tree was constructed using the bootstrap test.

**Figure 1:**
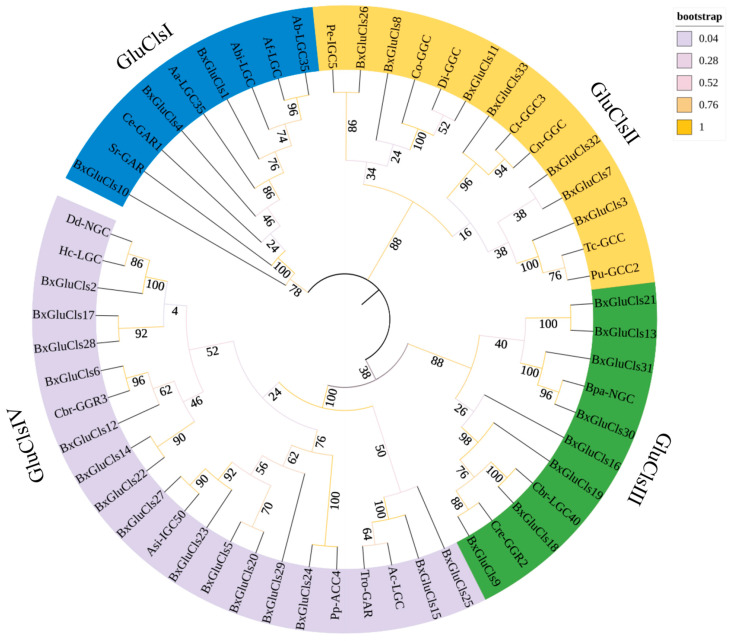
Phylogenetic tree of BxGluCls and GluCls in other nematodes. Note: Bx represents *Bursaphelenchus xylophilus*, Aa represents *Aphelenchoides avenae*, Abi represents *Aphelenchoides bicaudatus*, Ab represents *Aphelenchoides besseyi*, Ac represents *Ancylostoma caninum*, Af represents *Aphelenchoides fujianensis*, Asi represents *Anisakis simplex*, Bpa represents *Brugia pahangi*, Cbr represents *Caenorhabditis briggsae*, Ce represents *Caenorhabditis elegans*, Cn represents *Cylicocyclus nassatus*, Co represents *Cooperia oncophora*, Cre represents *Caenorhabditis remanei*, Ct represents *Cyathostomum tetracanthum*, Dd represents *Ditylenchus destructor*, Di represents *Dirofilaria immitis*, Hc represents *Haemonchus contortus*, Pe represents *Parascaris equorum*, Pp represents *Pristionchus pacificus*, Pu represents *Parascaris univalens*, Sr represents *Strongyloides ratti*, Tc represents *Toxocara canis*, and Tro represents *Trichostrongylus colubriformis*.

Based on the prediction results of the maximum likelihood tree in the best model, the LG+G+F model was selected to construct the phylogenetic evolutionary tree of the BxGluCls family proteins. This model had a BIC (Bayesian Information Criterion) value of 47,331.640 and an AICc (Corrected Akaike Information Criterion) value of 46,368.567. This model incorporates the LG substitution matrix, gamma-distributed rate heterogeneity, and empirical base frequencies. The model was chosen because it provided the lowest BIC value (47,331.640), indicating that it is the best fit for the data among the tested models. Bootstrap values (1000 replicates) were shown at the nodes, representing the statistical support for each branch. The different colors (e.g., blue, green, pink) represent different clades or groups of related sequences within the tree. These colors help to highlight the evolutionary relationships and groupings among the BxGluCls family proteins and their orthologs from other nematodes. Specifically, blue represents GluClsI family, yellow represents GluClsII family, and green and pink represents GluClsIII family and GluClsIV family, indicating distinct evolutionary lineages within the GluCls family (Figure 1).

The phylogenetic tree showed that the BxGluCls family proteins were divided into several major clades. GluCls1, GluCls4, GluCls10 of *B. xylophilus*, LGC35 of *Aphelenchoides besseyi*, LGC of *Ap. fujianensis*, LGC of *Ap. bicaudatus*, LGC35 of *Ap. avenae*, GAR1 of *Ca. elegans*, and GAR of *Strongyloides ratti* form a well-supported clade, suggesting a close evolutionary relationship. Similarly, GluCls2, GluCls5, GluCls6, GluCls12, GluCls14, GluCls15, GluCls17, GluCls20, GluCls22, GluCls23, GluCls24, GluCls25, GluCls27 of *B. xylophilus*, NGC of *Ditylenchus destructor*, LGC of *Haemonchus contortus*, GGR3 of *Ca. briggsae*, IGC50 of *Anisakis simplex*, ACC4 of *Pristionchus pacificus*, GAR of *Trichostrongylus colubriformis* and LGC of *Ancylostoma caninum* clustered together in another distinct clade, indicating potential gene duplication events within this group. GluCls3, GluCls7, GluCls11, GluCls26, GluCls32, GluCls33 of *B. xylophilus*, IGC5 of *Parascaris equorum*, GGC of *Cooperia oncophora*, GGC of *Dirofilaria immitis*, GGC3 of *Cyathostomum tetracanthum*, GGC of *Cylicocyclus nassatus*, GCC of *Toxocara canis*, and GCC2 of *Pa. univalens* were grouped together with high sequence similarity and bootstrap support, indicating that they may have arisen from a recent duplication event. Additionally, GluCls9, GluCls13, GluCls16, GluCls18, GluCls19, GluCls21, GluCls30, GluCls31 of *B. xylophilus*, NGC of *Brugia pahangi*, LGC40 of *Ca. briggsae*, and GGR2 of *Ca. remanei* form a separate clade with a relatively high number of exons, further supporting the idea of gene duplication and subsequent functional diversification. These clades were also supported by high bootstrap values, further validating their robustness.

**Figure 2:**
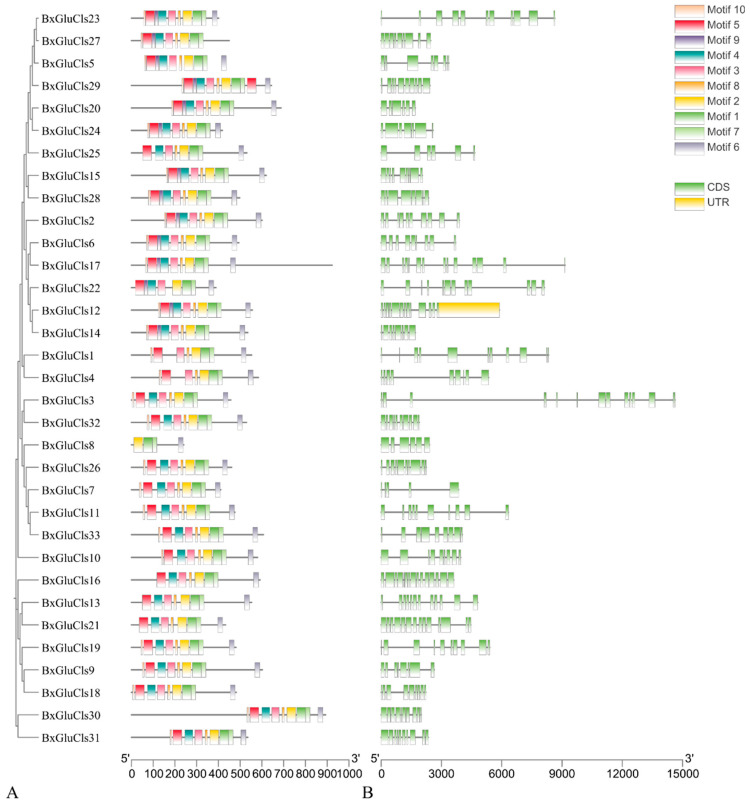
Comparison of the structure motifs distribution across the 33 BxGluCls proteins. Note: (**A**) Motif Distribution: This panel illustrates the distribution of conserved motifs across the 33 BxGluCls proteins. (**B**) Exon-Intron Structure: This panel shows the exon-intron structure of the *BxGluCls* genes, depicted in green. CDS (Coding Sequence): This refers to the portion of the gene sequence that is translated into the protein. It includes the exons and excludes the introns. UTR (Untranslated Region): These are regions of the mRNA that are not translated into protein. The 5’ UTR is located upstream of the coding sequence, while the 3’ UTR is located downstream. These regions can play important roles in gene regulation. Motifs: These are short, conserved sequences that often have functional significance. In the context of our study, the motifs refer to conserved sequence patterns within the BxGluCls proteins.

The compositions of the introns and exons in the BxGluCls family genes were determined by analysis. The results indicated that the number of exons in the 33 *BxGluCls* genes ranged from 5 to 14. Specifically, *BxGluCls*7 had only 5 exons, while *BxGluCls*16, *BxGluCls*17, and *BxGluCls*21 had the highest number of exons, totaling 14. A phylogenetic tree illustrates the evolutionary relationships among GluCls family genes from different nematode species, providing insights into the conservation and divergence of intron-exon structures. The analysis of structural motifs in the BxGluCls proteins revealed that these proteins shared similar motifs. The shared structural motifs were confirmed by the analysis of conserved motif structures (Figure 2). The figure illustrates the conserved motifs identified within the BxGluCls family, highlighting the regions of sequence similarity across the proteins. The clustering of these motifs indicated regions of high conservation, suggesting functional importance. The presence of 5’ and 3’ regions in the figure refers to the untranslated regions (UTR) of the gene sequences, while CDS denotes the coding sequence. These elements were included to provide a comprehensive view of the gene structure and to highlight the conserved motifs within the context of the full gene sequence.

### 2.4. Chromosomal Distribution of BxGluCls

**Figure 3:**
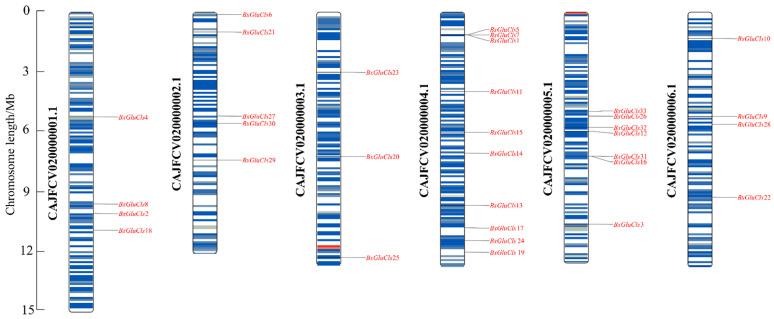
Chromosomal position of BxGluCls. Note: Each column represents a different chromosome, labeled from 1 to 6. The vertical axis represents the relative positions of genes along the chromosomes, with 5’ and 3’ regions indicated.

The chromosomal localization of the 33 *BxGluCls* genes in *B*. *xylophilus* was determined using TBtools v2.056, which identified the precise positions of these channel-encoding genes across different chromosomes (Figure 3). Chromosomal localization analysis revealed that most *BxGluCls* family genes were widely distributed in chromosomes. For example, *BxGluCls*4 was located in the middle of chromosome 1, while *BxGluCls*2, *BxGluCls*8, and *BxGluCls*18 were positioned in the 3′ region of chromosome 1. On chromosome 2, *BxGluCls*6 and *BxGluCls*21 were found in the 5′ region; *BxGluCls*27 and *BxGluCls*30 may be gene duplication events. However, *BxGluCls*20, *BxGluCls*23, and *BxGluCls*25 were dispersed on chromosome 3. On chromosome 4, *BxGluCls*1, *BxGluCls*5, and *BxGluCls*7 were located in the 5′ region, and tandem arrays may exist between them. *BxGluCls*11, *BxGluCls*14, and *BxGluCls*15 were in the central region; and *BxGluCls*13, *BxGluCls*17, *BxGluCls*19, and *BxGluCls*24 were positioned in the 3′ region. There were possible gene duplication events between *BxGluCls*26 and *BxGluCls*33, *BxGluCls*12 and *BxGluCls*32, *BxGluCls*16 and *BxGluCls*31, in the centromeric region of chromosome 5. *BxGluCls*9, *BxGluCls*10, *BxGluCls*22, and *BxGluCls*28 were located on chromosome 6. Notably, chromosome 4 contained the highest number of *BxGluCls* genes, followed by chromosomes 2 and 5, whereas chromosome 3 had the fewest. This distribution pattern suggests potential functional clustering or regulatory mechanisms associated with chromosomal architecture. These findings suggest that the *BxGluCls* family genes are not only widely distributed across chromosomes but also exhibit complex evolutionary dynamics, including tandem duplication and gene clustering.

### 2.5. Protein Structure Analysis of BxGluCls

**Figure 4:**
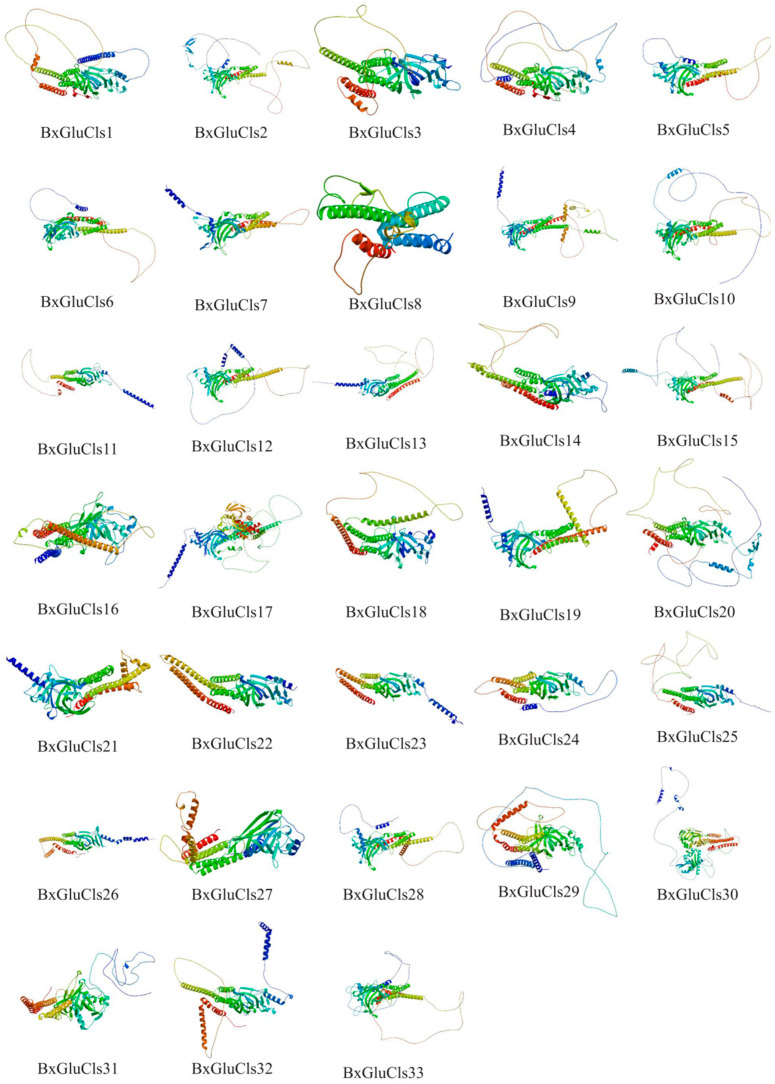
Predicted three-dimensional structures of BxGluCls, showing *α*-helices, *β*-folded structures, *β*-turns, and irregular(random) coils.

**Figure 5:**
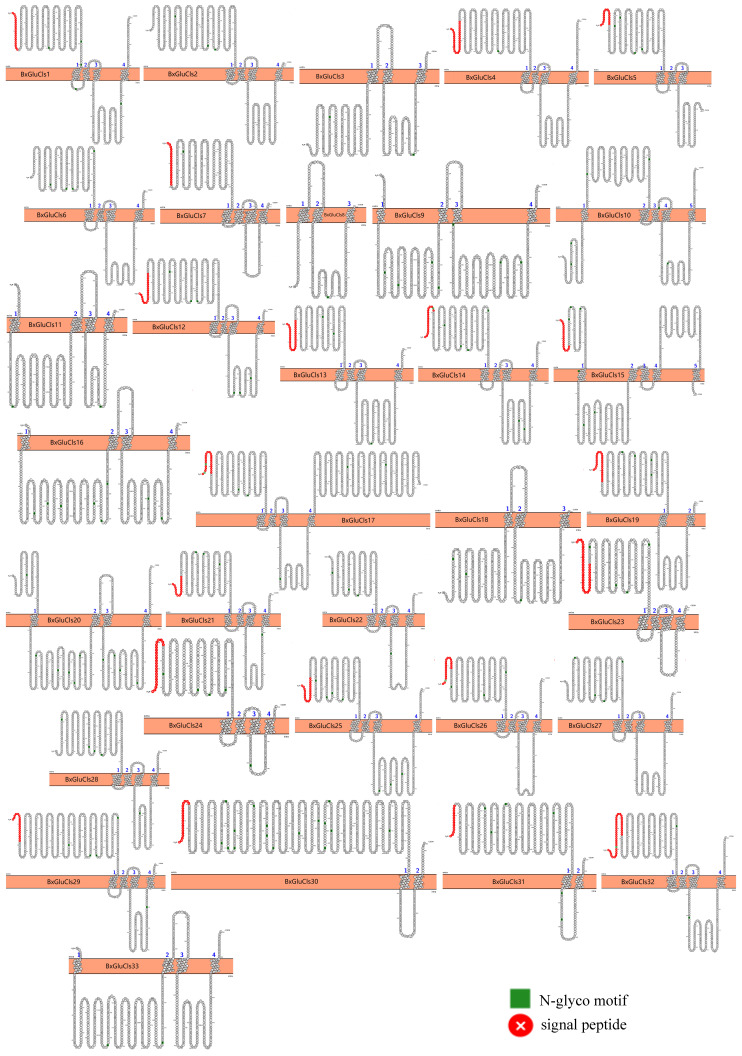
Predicted topologies of 33 BxGluCls proteins. Note: Glycosylation sites are indicated in green, the cell membrane is shown in orange, and signal peptides are highlighted in red.

Our analysis of the secondary structures of 33 BxGluCls proteins revealed that they had similar percentages of *α*-helices (21.46%–37.70%), *β*-folds (15.99%–27.51%), *β*-turs (1.50%–5.53%), and irregular coils (35.97%–59.16%). Moreover, through protein 3D structure prediction, we suggest that BxGluCls family proteins have small structural differences, i.e., their conformations are very similar. This implies that their functions are also similar. A detailed 3D structural analysis of BxGluCls proteins provides a comprehensive view of their key structural elements and functional domains. The high conservation of the pore region, ligand-binding pocket, and Cys-loop supports their classification as Cys-loop ligand-gated ion channels. These findings further validate our motif analysis and phylogenetic clustering results, highlighting the functional similarity and potential unique adaptations of BxGluCls proteins. The 33 BxGluCls proteins exhibited high sequence similarity. Despite this overall similarity, the number of potential N-glycosylation sites varied from one to 16 among the proteins, indicating functional diversity. Proteins with higher numbers of N-glycosylation sites may have additional roles in cellular signaling or structural stability (Table S1, Figure 4 and Figure 5).

### 2.6. Expression Profiles of BxGluCls in Different Stages

**Figure 6:**
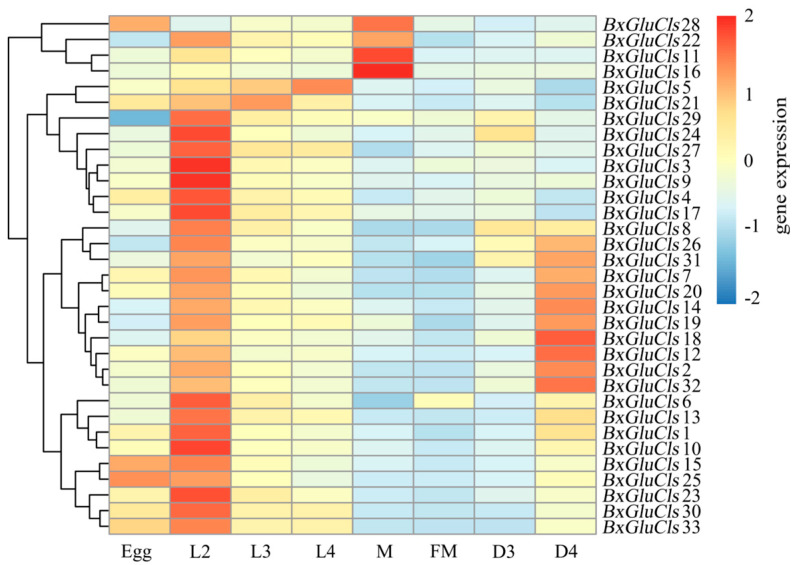
Expression pattern of *BxGluCls* in different ages. Note: Egg was egg stage of *B. xylophilus*; M was male adult of *B. xylophilus*; FM was female adult of *B. xylophilus*; L2 was second instar larva; L3 was third instar larva; L4 was fourth instar larva; D3 was dauer third-instar larva; D4 was dauer fourth-instar larva.

We were interested to see whether the gene expression of *BxGluCls* alters under different stages of the worm life cycle. To analyze gene expression in different developmental stages of *B. xylophilus*, we employed semi-quantitative reverse transcription polymerase chain reaction (RT-qPCR). We have also investigated the alterations in the gene expression of *BxGluCls* under the stress conditions. This approach allowed us to visualize the expression patterns of the 33 *BxGluCls* genes across different developmental stages and conditions. By analyzing gene expression data from *B. xylophilus* under various nematode states and stress conditions, the expression data of 33 *BxGluCls* genes were obtained. Heat map analysis (Figure 6) revealed significant variations in *BxGluCls* gene expression across different nematode states. Specifically, *BxGluCls*15, *BxGluCls*25, and *BxGluCls*28 were highly expressed in nematode eggs, suggesting their involvement in egg development. Except for *BxGluCls*5, *BxGluCls*11, *BxGluCls*16, and *BxGluCls*28, all other *BxGluCls* genes exhibited high expression in the second-instar larvae of adult *B. xylophilus*. Conversely, except for *BxGluCls*5, *BxGluCls*21, the expression of other *BxGluCls* genes were lower in the third and fourth-instar larvae during the growth period. The findings indicated that *BxGluCls* played a more significant role in the developmental process of *B. xylophilus* and had a reduced influence on the third and fourth instar larva. *BxGluCls*12, *BxGluCls*18, and *BxGluCls*32 were highly expressed in dauer fourth-instar larva (D4) *B. xylophilus*, suggesting their role in nematode resistance and adaptation to adverse environments. *BxGluCls*11, *BxGluCls*16, *BxGluCls*22, and *BxGluCls*28 were highly expressed in male adult nematodes (M), indicating their association with sex differentiation, particularly in male formation. Regarding the gene expression, the egg stage was totally different than the L2 stage, i.e., the first larvae stage. So, the transition from an egg to a larva was marked by a significant change in the expression pattern of 33 GluCls forms. L3 and L4 were quite similar, displaying no or very few visible changes in the *GluCls* gene expression pattern. However, when a larva became an adult, male or female, the transition was accompanied by a marked change in the gene expression pattern.

## 3. Discussion

Since its initial discovery in China in 1982, *B*. *xylophilus* has caused numerous major plant epidemics, spreading rapidly and posing a serious threat to China’s forestry production and ecological security [18]. Controlling *B. xylophilus* remains a significant challenge due to the emergence of pesticide resistance and environmental concerns associated with chemical treatments [19]. The invasion of alien species into new environments often leads to significant changes in gene families, which are crucial for their adaptation and evolution [20]. This study suggests that targeting GluCls could offer a novel and selective approach to managing PWD. Previous studies have demonstrated that GluCls are essential ion channels found exclusively in invertebrates, making them ideal targets for selective insecticides and nematicides. These channels are widely distributed in the nervous systems of nematodes, where they regulate various physiological processes, including movement, feeding, and sensation [21]. However, few studies have investigated the function of GluCls as an insecticide target in plant-parasitic nematodes [22]. A GluCls subunit database, based on information from 125 nematodes, has been published [23]. Additionally, the expression of *GluCls* genes has been shown to be up-regulated in *B. xylophilus* [24]. Therefore, elucidating the role of GluCls as a potential target for *B. xylophilus* is of paramount scientific and practical significance. In this study, bioinformatics analysis of the GluCls family in *B. xylophilus* provides valuable insights into its role in the nematode’s development, stress response, and potential as a target for novel biopesticides.

GluCls are known to play a critical role in neural signaling and muscle function in nematodes, with their selective expression patterns suggesting potential involvement in specific developmental processes. The expression dynamics of GluCls across developmental stages in *B*. *xylophilus* highlight their regulatory roles in nematode ontogeny, particularly in larval maturation and reproductive processes. The high expression of *BxGluCls*15, *BxGluCls*25, and *BxGluCls*28 in eggs indicates their potential contribution to early embryonic development. This finding aligns with studies in *C*. *elegans*, which demonstrate GluCls are essential for embryonic development and larval growth [25,26]. In nematodes, GluCls modulate ion gradients critical for oocyte maturation and embryonic viability, likely through calcium signaling pathways associated with eggshell formation [27]. Notably, the temporal specificity of these genes suggests stage-dependent regulatory mechanisms, potentially coordinated with hormonal cues such as ecdysteroids, which are pivotal in arthropod molting and may share conserved signaling cascades in ecdysozoans [28]. Similarly, the elevated expression of *BxGluCls12*, *BxGluCls18*, and *BxGluCls32* in dauer fourth-instar larva (D4) implies their role in resistance and adaptation to environmental stressors. The lower expression of some *GluCls* genes in adult stages suggests a diminished role in mature nematodes, consistent with findings in *Haemonchus contortus*, where certain GluCls subunits are predominantly expressed in larva [29]. Although sexual dimorphism in *B. xylophilus* remains underexplored, the differential expression of GluCls in larval stages hints at their potential involvement in sex-specific neural circuit maturation. The enrichment of *BxGluCls*25 in early larvae may influence gender-specific behaviors or gonad development, as observed in insects, where glutamate signaling regulates sex-determination pathways [30]. Future studies integrating single-cell transcriptomics could clarify whether specific GluCls isoforms are localized to reproductive tissues or sexually dimorphic neurons, providing insights into their roles in gametogenesis or mating behaviors.

Phylogenetic analysis revealed distinct clades within the BxGluCls family, indicating evolutionary conservation and divergence. Conserved motif analysis further highlighted the importance of specific structural elements for channel function, with variations in motifs potentially linked to functional adaptations [31]. This suggested that while some GluCls retain conserved functions, others have specialized roles in specific developmental stages or stress responses. The conserved structural features of BxGluCls proteins, as evidenced by their similar physicochemical properties and gene structure, suggested a high degree of functional conservation within this family. This conservation was consistent with the critical role of GluCls in neural signaling and muscle function across nematodes. The stage-specific expression patterns of *BxGluCls* genes further supported the hypothesis that these channels are involved in specific developmental processes and stress responses. The topological prediction of BxGluCls proteins revealed a canonical structure consistent with Cys-loop ligand-gated ion channels, with four transmembrane domains and extracellular N- and C-termini [32]. The unique features suggested potential functional adaptations specific to these proteins. These findings provided a structural basis for understanding the function and regulation of BxGluCls proteins.

By elucidating the structure, function, and expression patterns of *BxGluCls*, future research could focus on developing biopesticides that selectively target these channels, thereby reducing the survival and reproductive capabilities of *B.xylophilus*. This study provides a comprehensive characterization of the *BxGluCls* family in *B. xylophilus*, highlighting their conserved structural features and distinct expression patterns. These findings not only advance our understanding of the molecular mechanisms underlying the development and stress response of *B. xylophilus* but also offer valuable insights for the development of targeted control strategies. Furthermore, investigating the mechanisms responsible for the stage-specific expression and resistance-associated functions of *BxGluCls* genes could further clarify their role in the biology of *B. xylophilus*.

## 4. Materials and Methods

### 4.1. Identification of BxGluCls Family Members and Analysis of Their Physicochemical Properties

Firstly, we queried and downloaded the *GluCls* gene sequences of *Ap. bicaudatus*, *Ap*. *fujianensis*, *Ap*. *besseyi*, *Ap*. *avenae*, *Anisakis simplex*, *C. elegans*, *Brugia malayi*, *Ca. bimaculatus*, *Ca. teleostei*, *Ditylenchus destructor* and *Parascaris equorum* from NCBI database (https://www.ncbi.nlm.nih.gov/gene, accessed on 21 March 2025). Then we employed BLASTX to align the obtained *BxGluCls* gene sequences in *B. xylophilus* genome (PRJEB40022) by Wormbase (version: WBPS19) (https://parasite.wormbase.org, accessed on 21 March 2025) [33]. Then we characterized these candidate sequences used NCBI-conserved domains 3.21 (https://www.ncbi.nlm.nih.gov/Structure/cdd, accessed on 21 March 2025) [34] and InterPro 101.0 (https://www.ebi.ac.uk/ Interpro, accessed on 21 March 2025) [35]. This process helped to remove redundant gene sequences and obtain the protein sequences of BxGluCls. The physicochemical properties of BxGluCls were further analyzed by Expasy 3.0 (https://web.expasy.org/protparam, accessed on 21 March 2025) [36].

### 4.2. Phylogenetic Analysis of the BxGluCls Family and Analysis of Conserved Motifs

To elucidate the evolutionary relationships of BxGluCls, MEGA 11.0 software was employed to conduct multiple sequence alignments of 33 BxGluCls. The optimal model for the maximum likelihood tree was identified, and a maximum likelihood evolutionary tree was constructed, with the Bootstrap test performed 1000 times [37]. Furthermore, gene information for BxGluCls was extracted from the gene structure annotation file. Gene structure visualization and analysis were performed using the online tool GSDS 2.0 (http://gsds.gao-lab.org/, accessed on 21 March 2025) [38]. In parallel, MEME Suite 5.5.6 (https://meme-suite.org/meme/, accessed on 21 March 2025) [39] was used to analyze the distribution of conserved motifs in BxGluCls proteins, with the motif value set to 10 and other parameters set to default. The results from MEME were integrated with the maximum likelihood tree to generate gene structure maps annotated with conserved motif structures.

### 4.3. Chromosomal Distribution and Protein Structure Analysis of the BxGluCls Family

We screened the gene family members from gene structure annotation files. *BxGluCls* annotation information was extracted for information including chromosome location, start and termination sites, and gene length. Based on this information, a distribution density map of *BxGluCls* on *B. xylophilus* chromosomes was created by TBtools [40,41]. Subsequently, the secondary structure analysis of BxGluCls was conducted using online tools such as Prabi (last modification on September 2022, accessed on 21 March 2025) (https://doua.prabi.fr/software/cap3) [42], NetPhos 3.1 (https://services.healthtech.dtu.dk/services/NetPhos-3.1/, accessed on 21 March 2025) [43], and Protter 1.0 (http://wlab.ethz.ch/protter/start/, accessed on 21 March 2025) [44]. Additionally, Swiss-Model (https://swissmodel.expasy.org/interactive, accessed on 21 March 2025) [45] was utilized to predict the protein tertiary structure.

### 4.4. Expression Profiles of BxGluCls Family in Different Developmental Stages

To analyze gene expression in different developmental stages using semi-quantitative reverse transcription polymerase chain reaction (RT-qPCR), *B. xylophilus* at various developmental stages were synchronized. For embryos, synchronized eggs were obtained by placing mixed-stage *B. xylophilus* in petri dishes at 25 °C in the dark for 10 min. This allowed the eggs laid by pregnant females to adhere to the bottom due to surface glycoproteins. The upper layer of water and nematodes was carefully removed to collect the synchronized eggs. To elucidate the molecular response mechanisms of *BxGluCls* genes to different treatments, expression values of 33 BxGluCls were obtained from various insect states of *B. xylophilus*, including eggs. The resulting expression data were normalized and visualized using the Heatmap Plots tool in R-Studio 4.4.3 (http://cran.r-project.org, accessed on 21 March 2025) [46]. The Heatmap was constructed using the Complex Heatmap package in R, which supports hierarchical clustering with great flexibility. To obtain second instar larvae (L2), the synchronized eggs were placed in a food-free environment and allowed to hatch at 25 °C in the dark. The synchronized L2 were transferred onto a lawn of *B. cinerea* on a PDA plate. Nematodes were collected after 24, 48, and 72 h to obtain third instar larva (L3), fourth instar larvae (L4), and adults, respectively. The dauer third-instar larva (D3) and the dauer fourth-instar larva (D4) were isolated from infected pines. The male and female nematodes were obtained by manually picking them under a microscope using worm pickers.

## 5. Conclusions

In this study, we screened and characterized the *BxGluCls* family based on the GluCls family within the *B*. *xylophilus* genome, identifying 33 *BxGluCls* family members. Our analysis of the physicochemical properties of these proteins revealed that the number of amino acids ranged from 241 to 923, with molecular masses ranging from 27.29 to 105.70 kDa. As shown in Figure 3, a higher number of *BxGluCls* genes were distributed on chromosomes 2, 4, and 5. Gene structure and conserved motif analyses indicated that *BxGluCls* family members exhibited highly similar structures, suggesting evolutionary conservation within nematodes. Predictions of protein secondary and tertiary structures revealed minimal structural differences among *BxGluCls* family proteins, indicating functional stability and high synergy. We also analyzed the expression patterns of *BxGluCls* genes across various developmental stages of the nematode. These findings demonstrate that GluCls play a crucial role in the growth, development, and resistance mechanisms of *B. xylophilus*. This study provides a foundation for further research into the functional roles of *BxGluCls*. In summary, this study confirms that *BxGluCls* gene family members are essential for nematode embryonic development, growth, and sexual differentiation. However, their role in the immune processes of *B. xylophilus* requires further investigation.

## Figures and Tables

**Table 1:** Comparison of glutamate-gated chloride ion channels (GluCls) from *B. xylophilus* and those from other nematodes.

Protein ID of *B. xylophilus*	Protein Name	Sequence ID in NCBI	Protein Name in Other Nematodes	Other Nematodes Name	Scores of BlastP ^1^	Similarity *E*-Value ^2^
BXYJ5.040019200	BxGluCls1	KAI6177746.1	Ligand-Gated ion Channel	*Aphelenchoides bicaudatus*	683	0
BXYJ5.010220700	BxGluCls2	CRZ22203.1	BMA-AVR-14, isoform b	*Brugia malayi*	405	0
BXYJ5.050236700	BxGluCls3	KAI6190451.1	Ligand-gated ion channel 50	*Aphelenchoides bicaudatus*	796	0
BXYJ5.010094300	BxGluCls4	KAI6171507.1	Glutamate-gated chloride channel subunit beta	*Aphelenchoides bicaudatus*	575	0
BXYJ5.040019000	BxGluCls5	KAI1719268.1	Neurotransmitter-gated ion-channel ligand binding domain-containing protein	*Ditylenchus destructor*	596	0
BXYJ5.020004500	BxGluCls6	KAI1720087.1	Neurotransmitter-gated ion-channel ligand binding domain-containing protein	*Ditylenchus destructor*	555	0
BXYJ5.040019100	BxGluCls7	KAI1720087.1	Neurotransmitter-gated ion-channel ligand binding domain-containing protein	*Ditylenchus destructor*	555	0
BXYJ5.010211400	BxGluCls8	KAI6226376.1	Glutamate-gated chloride channel	*Aphelenchoides fujianensis*	247	9 × 10^−80^
BXYJ5.060084300	BxGluCls9	KAI6175995.1	Protein CBR-GGR-2	*Aphelenchoides bicaudatus*	721	0
BXYJ5.060015200	BxGluCls10	KAI6207390.1	Unc-49B protein	*Aphelenchoides besseyi*	766	0
BXYJ5.040069900	BxGluCls11	KAI6226375.1	BMA-AVR-14, isoform b	*Aphelenchoides fujianensis*	737	0
BXYJ5.050134500	BxGluCls12	KAI6192191.1	Ligand-Gated ion Channel	*Aphelenchoides bicaudatus*	647	0
BXYJ5.040223500	BxGluCls13	KAI6176054.1	Glycine receptor subunit beta-type 4	*Aphelenchoides bicaudatus*	701	0
BXYJ5.040164200	BxGluCls14	KAI1717202.1	Neurotransmitter-gated ion-channel ligand binding domain-containing protein	*Ditylenchus destructor*	644	0
BXYJ5.040127700	BxGluCls15	KAI6236273.1	Ligand-gated ion channel 50	*Aphelenchoides besseyi*	762	0
BXYJ5.050172600	BxGluCls16	KAI6199839.1	Glycine receptor subunit beta-type 4	*Aphelenchoides besseyi*	680	0
BXYJ5.040241100	BxGluCls17	KAI1719624.1	Neurotransmitter-gated ion-channel ligand binding domain-containing protein	*Ditylenchus destructor*	574	0
BXYJ5.010233100	BxGluCls18	KAI6173198.1	Ligand-Gated ion Channel	*Aphelenchoides besseyi*	570	0
BXYJ5.040257200	BxGluCls19	KAI1722739.1	Neurotransmitter-gated ion-channel ligand binding domain-containing protein	*Ditylenchus destructor*	532	0
BXYJ5.030152800	BxGluCls20	KAH7724515.1	CRE-LGC-46 protein	*Aphelenchoides avenae*	764	0
BXYJ5.020020400	BxGluCls21	KAI6171345.1	Glycine receptor subunit beta-type 4	*Aphelenchoides bicaudatus*	440	0
BXYJ5.060201900	BxGluCls22	KAI6179964.1	Cation transporter family protein	*Aphelenchoides besseyi*	482	2 × 10^−166^
BXYJ5.030045000	BxGluCls23	KAI6189254.1	ACC-1 protein	*Aphelenchoides besseyi*	583	0
BXYJ5.040248800	BxGluCls24	KAI6202533.1	Acc-4	*Aphelenchoides besseyi*	629	0
BXYJ5.030239900	BxGluCls25	KAI1716061.1	Neurotransmitter-gated ion-channel ligand binding domain-containing protein	*Ditylenchus destructor*	602	0
BXYJ5.050106900	BxGluCls26	QBZ81966.1	Ivermectin-sensitive glutamate-gated chloride channel subunit 5	*Parascaris equorum*	523	0
BXYJ5.020097700	BxGluCls27	AVV64053.1	Ligand-gated ion channel 50	*Anisakis simplex*	670	0
BXYJ5.060095300	BxGluCls28	KAI6189662.1	Cation transporter family protein	*Aphelenchoides bicaudatus*	627	0
BXYJ5.020169700	BxGluCls29	KAI6191605.1	Cation transporter family protein	*Aphelenchoides bicaudatus*	563	0
BXYJ5.020110300	BxGluCls30	KAI6193159.1	Glycine receptor subunit alphaZ1	*Aphelenchoides besseyi*	1200	0
BXYJ5.050172500	BxGluCls31	KAI1705856.1	Neurotransmitter-gated ion-channel ligand binding domain-containing protein	*Ditylenchus destructor*	604	0
BXYJ5.050127700	BxGluCls32	KAI1727299.1	Neurotransmitter-gated ion-channel ligand binding domain-containing protein	*Ditylenchus destructor*	732	0
BXYJ5.050098400	BxGluCls33	KAI6190334.1	Glutamate-gated chloride channel	*Aphelenchoides bicaudatus*	709	0

^1^ **BLASTP Scores**: BLASTP is a protein sequence alignment tool based on the BLAST 2.16 (Basic Local Alignment Search Tool) algorithm. Scores are important indicators for measuring the similarity between the query sequence and the target sequences in the database. ^2^ Similarity E-value: Similarity E-value is a statistical measure used to assess the significance of sequence alignment results.

**Table 2:** Physicochemical properties analysis of BxGluCls.

Protein Name	Number of Amino Acids	Molecular Weight /kDa	Isoelectric Point	Aliphatic Index ^1^	Hydropathicity ^2^	Instability Index ^3^	Stability
BxGluCls1	553	64.59	6.68	83.74	−0.188	51.19	Unstable
BxGluCls2	601	68.06	8.65	82.06	−0.176	50.16	Unstable
BxGluCls3	457	52.28	8.90	85.75	−0.030	38.30	Stable
BxGluCls4	584	66.25	6.58	90.15	−0.028	41.05	Unstable
BxGluCls5	448	51.56	7.46	88.30	0.023	39.46	Stable
BxGluCls6	494	56.97	8.66	89.90	−0.158	44.23	Unstable
BxGluCls7	411	46.70	5.66	98.32	−0.017	48.06	Unstable
BxGluCls8	241	27.29	9.76	103.53	0.101	47.60	Unstable
BxGluCls9	602	70.00	8.70	83.87	−0.403	41.37	Unstable
BxGluCls10	580	66.68	8.97	79.10	−0.289	45.70	Unstable
BxGluCls11	476	55.44	8.98	82.48	−0.224	47.19	Unstable
BxGluCls12	556	63.44	6.74	87.90	−0.220	46.22	Unstable
BxGluCls13	553	63.60	9.12	76.00	−0.296	56.18	Unstable
BxGluCls14	535	61.09	6.43	84.06	−0.226	53.42	Unstable
BxGluCls15	620	71.01	8.93	84.69	−0.161	41.35	Unstable
BxGluCls16	592	68.64	8.35	78.24	−0.250	50.47	Unstable
BxGluCls17	923	105.70	8.79	82.99	−0.272	37.64	Stable
BxGluCls18	482	55.83	6.50	81.35	−0.213	41.91	Unstable
BxGluCls19	481	55.51	9.02	84.74	−0.140	53.86	Unstable
BxGluCls20	688	78.86	8.55	77.02	−0.320	57.41	Unstable
BxGluCls21	433	50.49	5.78	88.24	−0.064	50.73	Unstable
BxGluCls22	389	45.71	8.76	94.42	0.020	39.73	Stable
BxGluCls23	401	47.40	8.84	95.79	0.064	40.84	Unstable
BxGluCls24	418	48.00	7.05	98.11	0.183	45.42	Unstable
BxGluCls25	531	60.66	8.70	78.17	−0.195	59.36	Unstable
BxGluCls26	461	53.17	9.38	95.14	−0.092	37.57	Stable
BxGluCls27	467	54.15	6.34	86.57	−0.049	46.27	Unstable
BxGluCls28	498	58.03	6.22	82.93	−0.216	54.99	Unstable
BxGluCls29	644	74.16	5.53	83.82	−0.212	48.58	Unstable
BxGluCls30	893	102.02	4.79	85.69	−0.370	42.28	Unstable
BxGluCls31	535	61.84	6.45	96.69	0.023	47.45	Unstable
BxGluCls32	529	61.57	9.12	89.91	−0.222	50.42	Unstable
BxGluCls33	590	67.24	9.08	82.58	−0.331	44.58	Unstable

^1^ Aliphatic index: The Aliphatic Index is a measure that describes the relative volume occupied by aliphatic side chains in a protein. ^2^ Hydropathicity: Hydropathicity is a relative value used to measure the hydrophobicity or hydrophilicity of a molecule. Positive values indicate hydrophobicity, while negative values indicate hydrophilicity. ^3^ Instability index: The Instability Index is a metric used to evaluate the stability of proteins. If the Instability Index is less than 40, the protein is considered stable. If the Instability Index is greater than 40, the protein is unstable.

## Data Availability

No data was used for the research described in the article.

## Supplementary Materials

The following supporting information can be downloaded at: https://www.mdpi.com/article/10.3390/ijms26083477/s1.
