# Supplementary material for: Bioinformatics Analysis of the Glutamate-Gated Chloride Channel Family in Bursaphelenchus xylophilus
Source: Int J Mol Sci. 2025 Apr 8;26(8):3477. doi: 10.3390/ijms26083477 (PMC12026476; doi:10.3390/ijms26083477)
Supplement: Supplementary file 1 [file ijms-26-03477-s001.zip › ijms-3486431-supplementary.pdf]

International Journal of Molecular Sciences

MDPI

Bioinformatics analysis of the glutamate-gated chloride channel family in *Bursaphelenchus xylophilus*

Table S1. Secondary structure motifs in BxGluCls.

Protein ID	Number of N-glycosylation sites	Number of amino acids							Percentage of number (%)			
		T	S	Y[1]	α-helix[2]	β-fold[3]	β-turn[4]	Random coil[5]	α-helix	β-fold	β-turn	Random coil
BxGluCls1	5	10	35	4	140	118	19	276	25.32	21.34	3.44	49.91
BxGluCls2	3	15	40	4	159	105	17	320	26.46	17.47	2.83	53.24
BxGluCls3	2	16	19	5	112	98	11	236	24.51	21.44	2.41	51.64
BxGluCls4	1	16	40	9	143	116	20	305	24.49	19.86	3.42	52.23
BxGluCls5	4	10	17	9	110	103	17	218	24.55	22.99	3.79	48.66
BxGluCls6	4	13	40	4	106	100	17	271	21.46	20.24	3.44	54.86
BxGluCls7	1	14	18	9	117	100	14	180	28.47	24.33	3.41	43.80
BxGluCls8	1	4	11	0	58	39	5	139	24.07	16.18	2.07	57.68
BxGluCls9	7	20	30	4	176	100	9	317	29.24	16.61	1.50	52.66
BxGluCls10	6	21	29	8	164	105	16	295	28.28	18.10	2.76	50.86
BxGluCls11	2	14	24	6	137	100	13	226	28.78	21.01	2.73	47.48
BxGluCls12	6	13	37	6	141	96	13	306	25.36	17.27	2.34	55.04
BxGluCls13	3	21	36	12	129	102	12	310	23.33	18.44	2.17	56.06
BxGluCls14	7	12	36	10	127	97	13	298	23.74	18.13	2.43	55.70
BxGluCls15	5	14	39	4	167	112	15	326	26.94	18.06	2.42	52.58
BxGluCls16	8	21	36	9	141	100	13	338	23.82	16.89	2.20	57.09
BxGluCls17	7	38	42	14	348	192	51	332	37.70	20.80	5.53	35.97
BxGluCls18	3	12	20	11	126	90	12	254	26.14	18.67	2.49	52.70
BxGluCls19	4	15	23	8	133	96	14	238	27.65	19.96	2.91	49.48
BxGluCls20	10	14	42	9	154	110	17	407	22.38	15.99	2.47	59.16
BxGluCls21	4	15	26	9	106	111	12	204	24.48	25.64	2.77	47.11
BxGluCls22	4	7	26	8	96	98	11	184	24.68	25.19	2.83	47.30
BxGluCls23	4	7	15	1	139	101	13	148	34.66	25.19	3.24	36.91
BxGluCls24	5	14	22	5	107	115	13	183	25.60	27.51	3.11	43.78
BxGluCls25	7	16	34	3	137	98	16	280	25.80	18.46	3.01	52.73
BxGluCls26	4	17	21	5	126	100	15	220	27.33	21.69	3.25	47.72
BxGluCls27	4	6	22	4	125	99	13	230	26.77	21.20	2.78	49.25
BxGluCls28	5	18	32	9	116	100	17	265	23.29	20.08	3.41	53.21
BxGluCls29	5	16	35	12	156	126	23	339	24.22	19.57	3.57	52.64
BxGluCls30	16	42	29	14	323	150	22	398	36.17	16.80	2.46	44.57
BxGluCls31	6	18	27	7	191	120	14	210	35.70	22.43	2.62	39.25

BxGluCls32	3	17	25	2	124	109	14	282	23.44	20.60	2.65	53.31
BxGluCls33	2	26	35	8	153	111	17	309	25.93	18.81	2.88	52.37

[1] T, S, Y: T is Threonine, S is Serine, and Y is Tyrosine, quantity share is the number of amino acids that make up each secondary structure as a percentage of the total number of amino acids.

[2] α-helix: The α-helix is a common secondary structure in proteins, formed by the right-handed coiling of a polypeptide chain around a central axis.

[3] β-fold: The β-fold is a secondary structure formed by the parallel or antiparallel alignment of polypeptide chains.

[4] β-*t*urn: The β-turn is a common type of turn structure, typically composed of four amino acid residues.

[5] Random coils: Random coil refers to the parts of a protein that cannot be classified as α-helices, β-fold, or other regular secondary structures.
